# Performance assessment of water reuse strategies using integrated framework of urban water metabolism and water-energy-pollution nexus

**DOI:** 10.1007/s11356-019-05465-8

**Published:** 2019-05-26

**Authors:** Oriana Landa-Cansigno, Kourosh Behzadian, Diego I. Davila-Cano, Luiza C. Campos

**Affiliations:** 1 Civil, Environmental and Geomatic Engineering, University College London, Gower St, London, WC1E6BT UK; 2 School of Engineering and Computing, University of West London, St. Mary’s Rd, London, W55RF UK; 3 Sistema Integrado de Tratamiento en los municipios de Rincón SITRATA, Camino a San Jerónimo s/n, col. San Jeronimo, 36407, Purisima, Guanajuato, Mexico

**Keywords:** Water-energy-pollution nexus, Urban water systems, Centralised and decentralised water reuse strategies, Urban water metabolism

## Abstract

This paper evaluates the metabolism-based performance of a number of centralised and decentralised water reuse strategies and their impact on integrated urban water systems (UWS) based on the nexus of water-energy-pollution. The performance assessment is based on a comprehensive and quantitative framework of urban water metabolism developed for integrated UWS over a long-term planning horizon. UWS performance is quantified based on the tracking down of mass balance flows/fluxes of water, energy, materials, costs, pollutants, and other environmental impacts using the WaterMet^2^ tool. The assessment framework is defined as a set of key performance indicators (KPIs) within the context of the water-energy-pollution nexus. The strategies comprise six decentralised water reuse configurations (greywater or domestic wastewater) and three centralised ones, all within three proportions of adoption by domestic users (i.e. 20, 50, and 100%). This methodology was demonstrated in the real-world case study of San Francisco del Rincon and Purisima del Rincon cities in Mexico. The results indicate that decentralised water reuse strategies using domestic wastewater can provide the best performance in the UWS with respect to water conservation, green house gas (GHG) emissions, and eutrophication indicators, while energy saving is almost negligible. On the other hand, centralised strategies can achieve the best performance for energy saving among the water reuse strategies. The results also show metabolism performance assessment in a complex system such as integrated UWS can reveal the magnitude of the interactions between the nexus elements (i.e. water, energy, and pollution). In addition, it can also reveal any unexpected influences of these elements that might exist between the UWS components and overall system.

## Introduction

The integral management of urban water systems (UWS) is primarily recognised for addressing services to water supply, stormwater and wastewater collection, and treatment within urban areas. The quality of the services provided in UWS can be evaluated by a number of performance criteria within the framework of sustainability (Behzadian and Kapelan 2015a). More specifically, UWS services should ideally act in such a way as to fulfil the technical, environmental, social, and economic requirements of sustainability aspects. For example, while it must provide the highest reliability to satisfy customers (i.e. social aspects), the adverse environmental impacts such as GHG emissions and pollutants discharge into receiving water bodies should be minimised. This performance is likely to be affected by some external scenarios such as urbanisation, population growth, and climate change. As a result, due to increasing water demands and more pressure on limited water resources, more attention must be paid to providing alternative water sources such as greywater or reclaimed water from water reuse options. In addition, water reclamation and reuse options are fundamental for city development and the reinforcement of circularity within the economy, which encompasses closing loops in material and energy flows, and minimising resource inputs and outputs for more efficient processes in cities (Geissdoerfer et al. 2017). Strategic implementation of water reuse is also important to fulfil political agendas towards sustainable development goals (WWAP 2017).

Among the water reuse options, it could be argued that centralised water reuse is more common in urban areas. Reclaimed water as a result of the treated effluent in centralised wastewater treatment works (WWTW) can be used for different demands including irrigation and toilet flushing in cities (Jiménez-Cisneros 2014). This approach benefits from the economy of scale although it is difficult to implement in rapidly urbanised cities due to space and resource constraints. On the other hand, decentralised water reuse is gaining more attention in cities for its modular design that is implementable near the source of generation such as households, high-rise buildings, or parts of a city in response to demand (Novotny 2013; Bieker et al. 2010). Wastewater in decentralised reuse strategies can generally be divided into greywater (effluent from the shower, washing machine, hand basin, dishwasher and kitchen) and black water (urine and faeces; Larse et al. 2016; Friedler et al. 2013; Domènech 2011). Existing reuse guidelines recommend effluent concentrations of biochemical oxygen demand (BOD) < 30 mg/L for use in urban irrigation or toilet flushing, which require treatments up to tertiary level (EPA 2012). Although wastewater treatment technologies seem to be highly efficient, they might also be energy intensive or produce more carbon emissions depending on the scale. Hence, the comparison of centralised vs decentralised water reuse is still an ongoing debate requiring further study (Chang et al. 2017; Valek et al. 2017; Matos et al. 2014; Mo et al. 2014; Opher and Friedler 2016; Singh et al. 2016).

Due to the widespread use of systemic assessment approaches for the analysis of water reuse alternatives, the comprehension of technical and environmental implications of UWS performance has increased significantly (Chen et al. 2012). The water-energy (WE) nexus assessment framework is a recently used type of systemic approach that highlights the linkages between water and energy and sometimes their connection with other sectors such as water-energy-food, water-energy-climate, or water-energy-pollution (WEP). Multiple frameworks and approaches to nexus assessment have been suggested by researchers in recent decades. Some studies focused on the nexus of energy and carbon footprints for comparative analysis among different wastewater treatment technologies (Singh and Kansal 2018; Gu et al. 2016; Singh et al. 2016) or various UWS at city scale (Valek et al. 2017). Other research works have used scenario analysis in the nexus framework to estimate the potential of water and energy savings in UWS when implementing greywater or rainwater strategies. Such scenarios are mostly calculated through material flow analysis (MFA) or input and output (IO) methodologies (Silva-Vieira and Ghisi 2016; Duong et al. 2011). There has been consistent growth in extending the WE nexus to other areas, such as environmental assessments, with considerably greater focus on GHG emissions. Such frameworks included life cycle assessment (LCA) (Opher and Friedler 2016; Lane et al. 2015; Mo et al. 2014) or ecological network analysis (ENA) (Wang and Chen 2016). A few studies have demonstrated this nexus framework through system dynamics by using the casual relationship of the water sector to energy and costs from a residential end-use perspective (De Stercke et al. 2018) or from the food sector at national level (Sušnik 2018). Another nexus assessment approach was suggested through optimisation models to obtain trade-offs between nexus elements (Tsolas et al. 2018; Zhang and Vesselinov 2016). The WEP nexus in the UWS is defined here as the linkages between water, energy, and pollutant loads in the main components of the UWS during its operational phase (Kumar and Saroj 2014). The analysis of the WEP nexus in UWS can lead to reducing pollutants discharged into receiving water bodies while saving the energy used for removing pollutants in the treatment stage (Chang et al. 2017; Kumar and Saroj 2014). This can be carried out by some available tools that can concurrently model water flows and pollutant loads such as GloWPa and WorldQual at catchment scale (Kroeze et al. 2016), and UVQ (Urban Volume and Quality; Mitchell and Diaper 2005) and WaterMet^2^ (Behzadian and Kapelan 2015a) at UWS level, to mention but a few. However, none of these models has extensively been used for comparing water reuse strategies within the assessment framework of the WEP nexus.

Another integrated analysis of the UWS is conducted through urban water metabolism derived from the urban metabolism approach proposed by Wolman (1965). This uses the analogy of city as living organism, in which both will demand input flows (such as energy and fuel), produce outputs (such as waste, emissions, and pollutants), and recycle waste for self-consumption. As water mass balance is dominated in cities (Wolman 1965), water metabolism research has been suggested as a priority for cities (Kenway 2013). Urban water metabolism specifically refers to the capacity and services in UWS, and their metabolic performance is analysed through various flows and fluxes of water, energy, materials, and other environmental impact categories within the system over a specific time period (Behzadian and Kapelan 2015a; Huang et al. 2013).

The theory and concept of urban water metabolism includes a wide range of performance implications, changing from resource efficiency and hydrological performance of different water servicing options in Australia (Farooqui et al. 2016), to ecological relationships among different UWS sectors and their wastewater discharges in China (Zheng et al. 2019). The integration of urban metabolism and the nexus approaches for performance assessment in real-world systems has not been employed substantially. More specifically, to the best of the authors’ knowledge, there are limited applications of the urban metabolism concept to understand the linkages between water and energy in cities. For example, Kenway (2013) quantified the connection between water and energy flows through the urban metabolism approach of four Australian cities. However, the urban metabolism framework has not yet been used for assessment of the WEP nexus. This study aims to explore the impact assessment of centralised and decentralised water reuse strategies in integrated UWS using the integrated assessment framework of urban water metabolism and the WEP nexus. The general scheme of the integrated framework developed in this study comprises two main components: an integrated UWS modelling for the simulation of the sustainability performance of water reuse strategies, and a set of performance indicators for WEP nexus assessment. The next section describes the methodology used in this paper, followed by its demonstration in a real case study in Mexico. Then, results and discussion are presented, followed by conclusions and future recommendations.

## Methodology

### Assessment framework

The proposed framework comprises an urban water metabolism model (i.e. WaterMet^2^) coupled with a set of key performance indicators derived from the WEP nexus for a performance assessment of water reuse strategies in an integrated UWS. The WaterMet^2^ model (Behzadian et al. 2014a) is customised here to analyse various centralised and decentralised water reuse strategies. The basic concepts and input data requirements in WaterMet^2^ are briefly outlined below.

#### WaterMet^2^ model

WaterMet^2^ (WM2) is a conceptual mass balance-based model for the simulation of the metabolic performance of an integrated UWS. WM2 is a dynamic MFA (mass flow analysis) model with daily time step simulation combined with an environmental impact assessment for the long-term duration (e.g. 20–40 years). WM2 aims to evaluate the metabolic performance of UWS for business-as-usual (BAU) and any water management strategies.

**Fig. 1:**
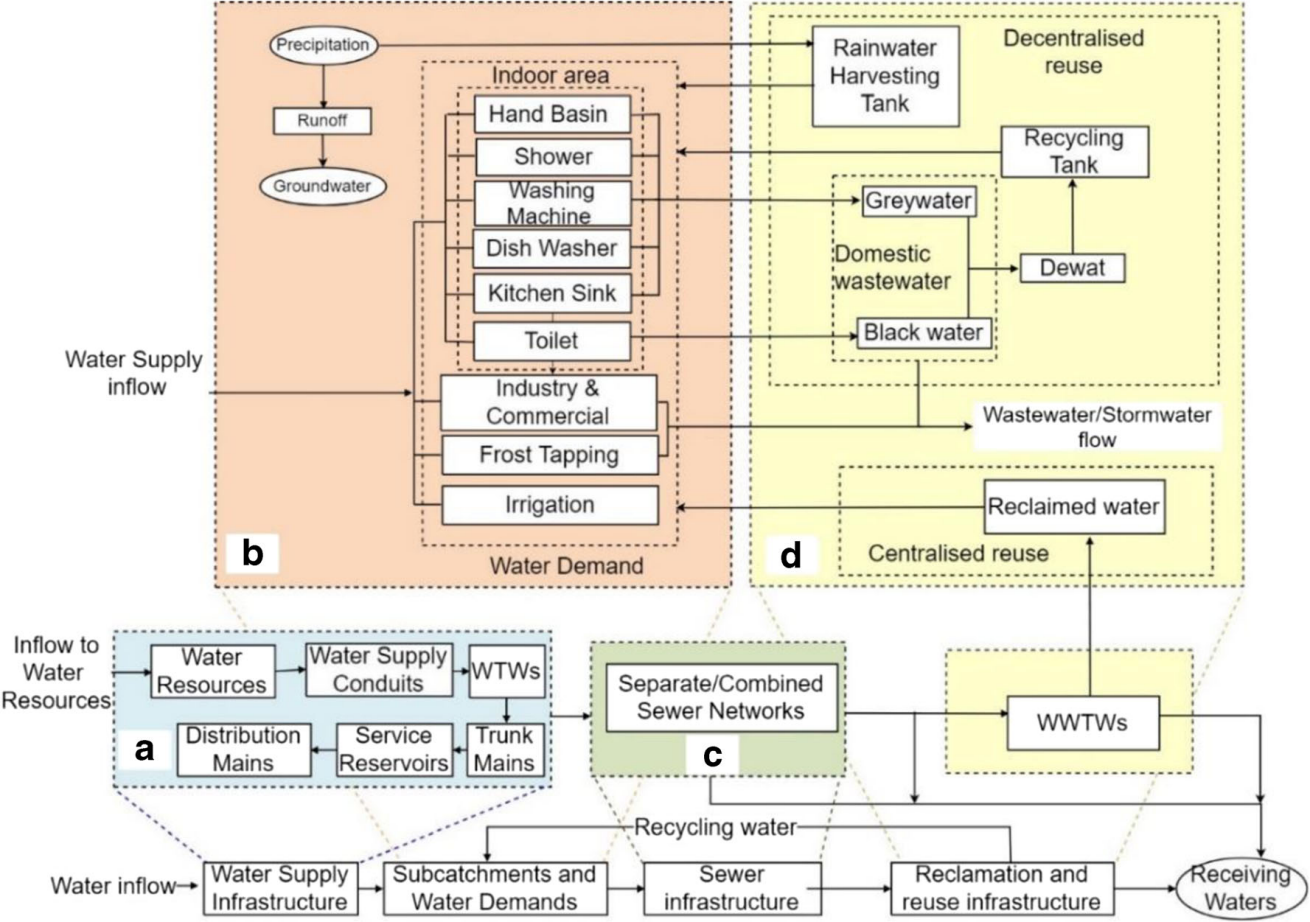
UWS main subsystems and components in WM2: (**a**) potable water supply, (**b**) subcatchment, (**c**)sewerage, and (**d**) water resource recovery. Modified from Behzadian and Kapelan (2015a)

The main UWS components in WM2 is included in four subsystems (Fig. 1): (a) *potable water supply* with components of water resources, water supply conduits, water treatment works (WTW), trunk mains, service reservoirs, and distribution mains; (b) *subcatchment* with components of local areas in which water demand profiles and rainfall-runoff parameters are defined; (c) *sewerage* with components of stormwater and wastewater collection networks, wastewater treatment works (WWTW), and receiving water bodies; and (d) *water resource recovery* with two components of centralised and decentralised facilities in which rainwater, greywater, or wastewater is collected, treated, and transported/distributed to water demand points. WM2 is a distributed model which means any number of these components can be modelled in UWS. Water reuse in centralised water reuse is transported from WWTWs while decentralised water reuse takes domestic wastewater (i.e. a mix of greywater and black water) or greywater alone to be treated and collected within decentralised wastewater treatment systems (DEWATS) in local areas. The remaining wastewater not considered for reuse purposes is discharged into sewer networks and, eventually, receiving water bodies.

The model tracks down the main flows and fluxes of water, energy, materials, chemicals, pollutants, and other environmental impact categories through the main UWS components. More specifically, the model simulates daily time step of water flows in the UWS components in various forms, i.e. green water from rainwater and blue water from surface/ground water in the water supply subsystem; and greywater and black water from water consumption in the sewerage subsystem. The domestic sewage and stormwater collected in sewer networks are simulated on a daily basis and are transported to WWTWs where the influent is treated based on pollutant removals. The treated effluent is then either discharged into receiving water bodies or returned to water reuse applications. A simplified approach for water quantity modelling is adopted in WM2 by using a daily mass balance of the water flows without any travel time of water quality routing. Hence, sequential daily water quality modelling allows for the tracking of any contaminant loads. WM2 tracks down the daily pollution loads of any pollutant defined by the user based on the complete mixing assumptions at any UWS components (Mitchell and Diaper 2005). Such a simplification is inevitably considered for other similar conceptually based models, including the Urban Water Optioneering Tool (UWOT) developed by Makropoulos et al. (2008) and UVQ produced by Mitchell and Diaper (2005). The model requires daily water demand profiles and their temporal variations (daily/monthly/annually) over the planning horizon due to seasonal and annual fluctuations (Venkatesh et al. 2017). The daily and monthly variations can be adjusted by using historic data through the process of model calibration, while the yearly variations can be set based on the projections of population growth scenarios. The daily capacity of storage, transportation, and treatment is the input data for the simulation of water flow in the main UWS components through the balancing of stocks and flows (Behzadian et al. 2014a).

WM2 then calculates quantitative key performance indicators (KPIs) related to various aspects of the sustainability framework such as economic, social, and environmental factors (Behzadian and Kapelan 2015b). The model also calculates a number of KPIs related to environmental impact categories similar to those in LCA such as eutrophication, acidification, and GHG emissions. These KPIs are mainly calculated by multiplying the simulated water and energy flows by corresponding factors. Unlike the LCA, KPIs in WM2 can be calculated spatially for individual components or the entire UWS and temporally for each time step and aggregated for larger periods. The metabolism performance simulation and corresponding KPIs in WM2 are limited to the operational stage of the UWS as construction, maintenance, and demolition phases have a small impact on environmental impact analyses compared to the operational phase (Jeong et al. 2015; Lane et al. 2015). That being said, all impacts of fabrication and transportation of materials and chemicals in the operational phase are considered in WM2. The spatial limit of the UWS in WM2 is the administrative limits of an urban water utility. The input data in WM2 are divided into three sections of time series, the UWS components, and other associated data. The time series data include daily inflows to water resources and daily weather data. The data required in UWS components are defined connections (i.e. topology) between the components, their operation in each subsystem and capacity for storage (i.e. water resources and service reservoirs), treatment (i.e. WTWs and WWTWs), and conveyance (i.e. water supply conduits, trunk/distribution mains, and sewerage). Other necessary information for operation is also defined here in UWS components such as energy and chemicals used, operational costs, leakage, and removal efficiency of pollutants. Four spatial scales of indoor, local area, subcatchment, and city are modelled in WM2 (Behzadian et al. 2014b). Water demand profile and energy uses for appliances and fittings are defined at household level, while the local area level defines commercial and outdoor water demands (i.e. garden watering and irrigation) as well as parameters for rainfall-runoff modelling. The specifications of decentralised water resource recovery facilities such as rainwater harvesting and greywater recycling tanks are defined at both local area and subcatchment levels. These specifications include storage capacity, costs, energy use, pollutant removal efficiency, and source/sink of water reuse. The average concentration of pollutants at household/industrial level and various urban surfaces (i.e. roof, road, pavement, and pervious areas) at local area level are defined as other input data. The factors required to calculate KPIs such as embodied energy, GHG emissions, and other environmental impacts are the secondary data used in WM2 that can be taken from the relevant literature. Further details of this information on WM2 can be found in Behzadian et al. (2014b).

WM2 was chosen here among similar tools due to its capacity to quantify various flows such as water, energy, and environmental impacts simultaneously through a metabolism framework. For instance, UWOT (Makropoulos et al. 2008) and UVQ (Mitchell and Diaper 2005) are demand-oriented approaches at different spatial scales and proven tools for water recycling modelling. However, they are mainly limited to a couple of impact categories (water and energy) and cannot consider the entire main UWS components in the modelling of the urban water cycle. WM2 has been used for multiple purposes such as water recycling (Behzadian et al. 2018) and reliability/resilience assessment (Morley et al. 2016) and demonstrated in several case studies. For example, it has been used for comparison in rainwater harvesting, greywater recycling, and desalination scenarios under population growth on the Galapagos Island, Ecuador (Reyes et al. 2017); for optimisation of non-conventional water source schemes to minimise water demand and reduce local flooding in Oslo (Behzadian et al. 2018); and in support of the decision analysis of increased water sources or pipeline rehabilitation in European cities (Morley et al. 2016; Behzadian and Kapelan 2015b). More recently, it has been used to compare centralised and decentralised water reuse options (Landa-Cansigno et al. 2018).

#### Key performance indicators

A set of five KPIs, as shown in Table 1, was rigorously selected from the three angles of the WEP nexus to carry out the performance assessment of water reuse strategies. The definition and assumptions of these KPIs are outlined here: (a) *Reliability of water supply* is defined as the ratio of the total water supplied to the total water demand over the planning horizon and is expressed in percentage (Behzadian and Kapelan 2015a). Hence, fully supplied water demands have a reliability of 100% and any reliability less than that indicates a lack of water supply over the planning horizon; (b) *Potable water* is defined as the amount of potable water supplied from conventional water resources. Note that the total water supplied is the sum of potable water and reuse water used to fulfil water demands in UWS; (c) *Net energy* is the result of balancing both consumed (i.e. caused) and avoided energy in the UWS components. Consumed energy includes both direct energy used from fossil fuels/grid electricity and indirect/embodied energy obtained from chemicals and materials used in the operational phase. The avoided energy includes renewable energy as electricity produced from biogas combustion in WWTWs and the embodied energy retrieved from resource recovery in WWTWs; (d) *GHG emissions*, expressed in kg CO_2_-eq, are calculated as the direct and indirect CO_2_ emitted from the UWS components, plus fugitive emissions of CO_2_, methane (CH_4_), and nitrous oxide (N_2_O) in WWTWs. The direct emissions include burning fossil fuels and those used for mains electricity generation, while indirect emissions are comprised of those used for embodied energy in chemicals, materials, and resource recovery. The factors used for conversion to kg CO_2_-eq are 28 for CH_4_ and 265 for N_2_O, according to the IPCC (2014); (e) *Eutrophication potential (EP)*, expressed in kg Phosphates equivalent (PO_4_-eq), is calculated based on the direct presence of phosphorus (P), nitrates (NO_3_), and chemical oxygen demand (COD) in effluents, emissions of ammonia (NH_3_) in sludge management, and indirect impact caused by the production of electricity, fossil fuel, chemical, and sludge disposal (Behzadian et al. 2014b). The conversion factors to PO_4_ are 0.35 for NH_3_, 0.022 for COD, and 3.06 for P (Heijungs et al. 1992).

**Table 1:** Description of key performance indicators

Nexus component	KPI	Unit
Water	Reliability of water supply	%
	Potable water	m^3^/year
Energy	Total energy	kWh/m^3^/year
Pollution	GHG emissions	kg CO_2_-eq/m^3^/year
	Eutrophication potential (EP)	Kg PO_4_-eq/m^3^/year

The pollution (Table 1) is estimated here based on the load mass balance of the following pollutants: COD, Total Suspended Solids (TSS), Total Nitrogen (TN), and Total Phosphorus (TP), using user-input concentrations (mg/L). These are defined here through average values from the literature review. Pollutant mass flows are tracked down within subcatchments and wastewater components in UWS. Removal of pollutants from a conventional activated sludge considered for this study was 91% BOD, 97% TSS, 94% COD, and 60% TN and TP (SITRATA 2017). The changes to four KPIs (i.e. potable water, energy, GHG emissions, and EP) in water reuse strategies are evaluated with respect to business-as-usual (BAU) (i.e. ‘do nothing’ over the planning horizon). Hence, these KPIs are also presented as a percentage of change relative to the BAU.

### Case study

**Fig. 2:**
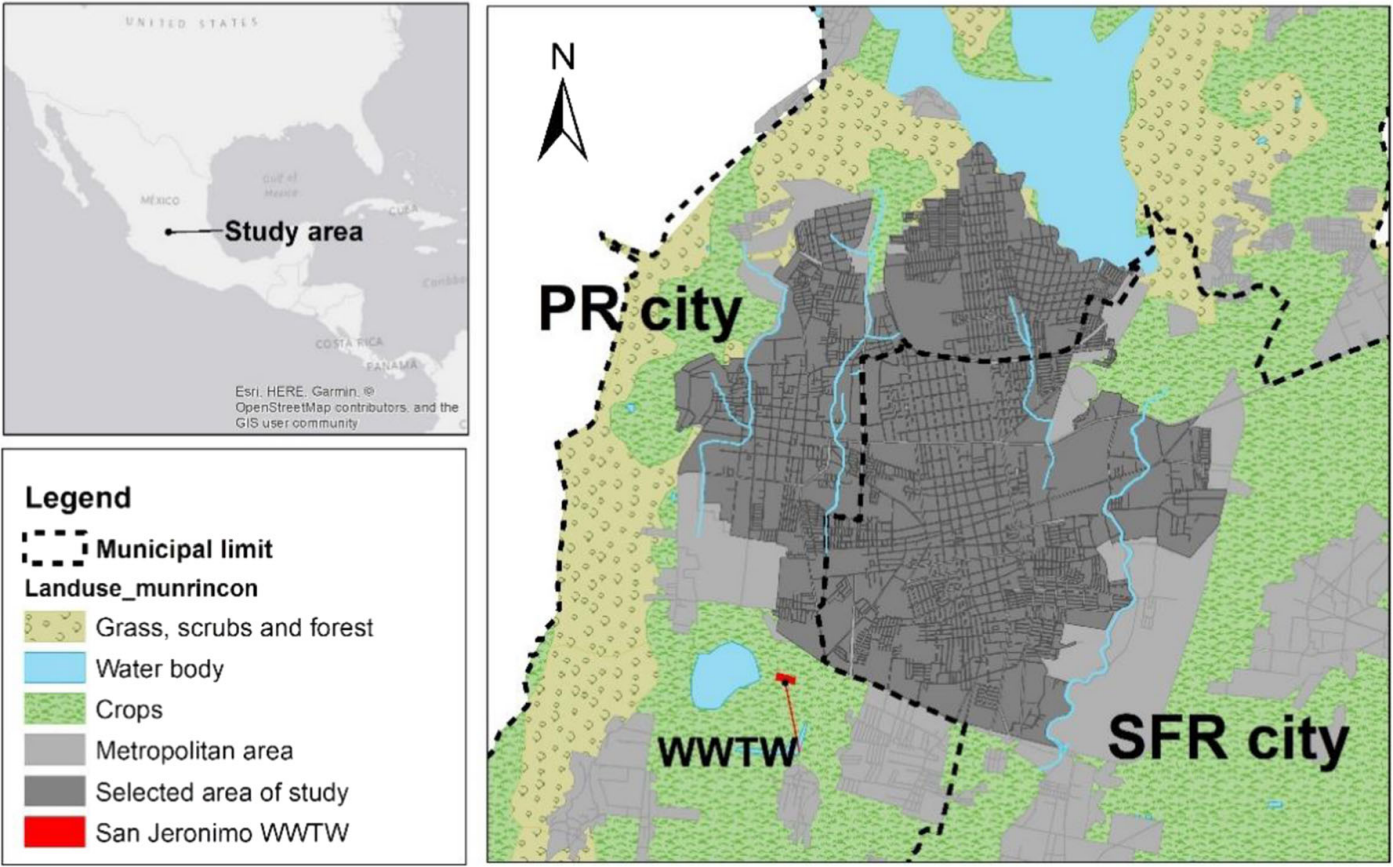
Location of the case study

The suggested methodology was demonstrated in a real-world case study in the metropolitan area formed by San Francisco del Rincon (SFR) and Purisima del Rincon (PR) cities, Guanajuato in Mexico (Fig. 2).

The UWS of this case study is one of the few urban areas planned for water reuse in the country with the following description. There are 22 boreholes at depths of 70–300 m (SAPAF 2017) withdrawing groundwater from Turbio Aquifer. This is the only water supply for the area and requires onsite chlorination according to the national potable water guideline (NOM-127-SSA1 1994). Potable water is stored in elevated tanks and then distributed by gravity to consumers with considerable leakage, i.e. 40–50% (CEAG 2014). Potable water is used for domestic, industrial, and public sectors in both cities. The domestic sector has 114,150 inhabitants spread over 31,261 households, but only 80% (24,751) of the houses have a potable water service, thus the population served numbers 111,600 only (INEGI 2010). This sector demands 89% and 71% of the total potable water in PR and SFR, respectively. Daily consumption per capita varies from 90 to 180 L/day in the region (CEAG 2017). The industrial and commercial sectors are composed of more than 2000 businesses of various sizes. Some are as small as food stalls with 2–4 workers and as large as shoe or automobile component manufacturing businesses with more than 150 workers (DENUE 2015). In total, they account for 8% and 23% of the total water demand in PR and SFR, respectively. The public sector includes water demands in hospitals, schools, and the irrigation of parks, sports facilities, and green lanes. Public demand accounts for 2.7% in PR and 5.4% in SFR (CEAG 2017). Although these percentages are reported for the entire municipality, it was assumed to be equal for the cities. The water demand flows per sector were calculated here by multiplying such percentages per water withdrawal flows after leakages.

Wastewater discharged into the sewerage network covers 99% of urban households that also receive potable water. Sewerage is a combined network with a total capacity of 43,200 m^3^/day. Each city has individual sewer networks, but both discharge the wastewater into “San Jeronimo” WWTW with an average treatment capacity of 21,600 m^3^/day. The overflows from the sewer network and WWTW discharge into the Turbio River as receiving water. The WWTW uses an activated sludge treatment coupled with screening and degritting, primary and secondary clarifiers, and a disinfection stage using chlorination or UV lamps. The plant was designed to treat the effluent at a quality of 30 mg BOD/L in order to comply with the non-potable water reuse guideline in Mexico (NOM-003-SEMARNAT 1997). Reclaimed water is mainly distributed for urban irrigation and soil compaction through trucks with a tank capacity of 20 m^3^. Water reuse reached only 1% of the wastewater inflow in 2015. This estimation might increase due to the construction of reclaimed water networks with a capacity of 250 m^3^/day. An anaerobic digester stabilises the sludge and produces biogas at a rate of 47.5 m^3^/h. The biogas is stored in a pressure container from which 40% of the total volume produces electricity at a rate of 0.03 kWh/m^3^ (SITRATA 2017). The remaining biogas is burned before being released into the atmosphere. Dewatered stabilised sludge is often deposited into agricultural fields nearby, with the remaining sludge and grit waste transported to landfill. A summary of the main UWS characteristics is shown in Table 2.

**Table 2:** Summary of main UWS characteristics of the case study

		Total	SFR	PR	Reference
Local area	Area (ha)	2844	1615	1229	INEGI (2016)
	Pervious area (%)	–	26	31	
	Impervious area (%)	–	58	56	
	Roof area (%)	–	16	13	
	Population served (inhabitants)	111,600	69,169	42,431	INEGI (2010)
	Households with access to potable water (number)	24,960	15,614	9344	
	Average occupancy per household	–	4.43	4.54	
Water resource	Water boreholes (Qty.)	22	12	10	SAPAF (2017) SAPAP (2017)
	Annual withdrawals in 2015 (× 10^3^ m^3^/y)	9477	6105	3372	
	Energy (kWh/m^3^)	–	0.40	0.44	
	Leakages (%)	–	40	53	CEAG (2014)
Annual water demand	Total water demand (× 10^3^ m^3^/y)	5451	3843	1608	CEAG (2017) SAPAF (2017) SAPAP (2017)
	Domestic demand (× 10^3^ m^3^/y)	4166	2734	1432	
	Public use and irrigation (× 10^3^ m^3^/y)	251	207	44	
	Industrial demand (× 10^3^ m^3^/y)	1034	902	132	
Sewer networks	Transportation capacity (m^3^/day)	47,300	–	–	SITRATA (2017)
Wastewater and resource recovery	Plant capacity (m^3^/day)	21,600	–	–	
	Energy use (kWh/m^3^)	0.38	–	–	
	Biogas production (m^3^/h)	47.5	–	–	
	Energy production (kWh/m^3^)	0.03	–	–	

### Model setup and input data

**Fig. 3:**
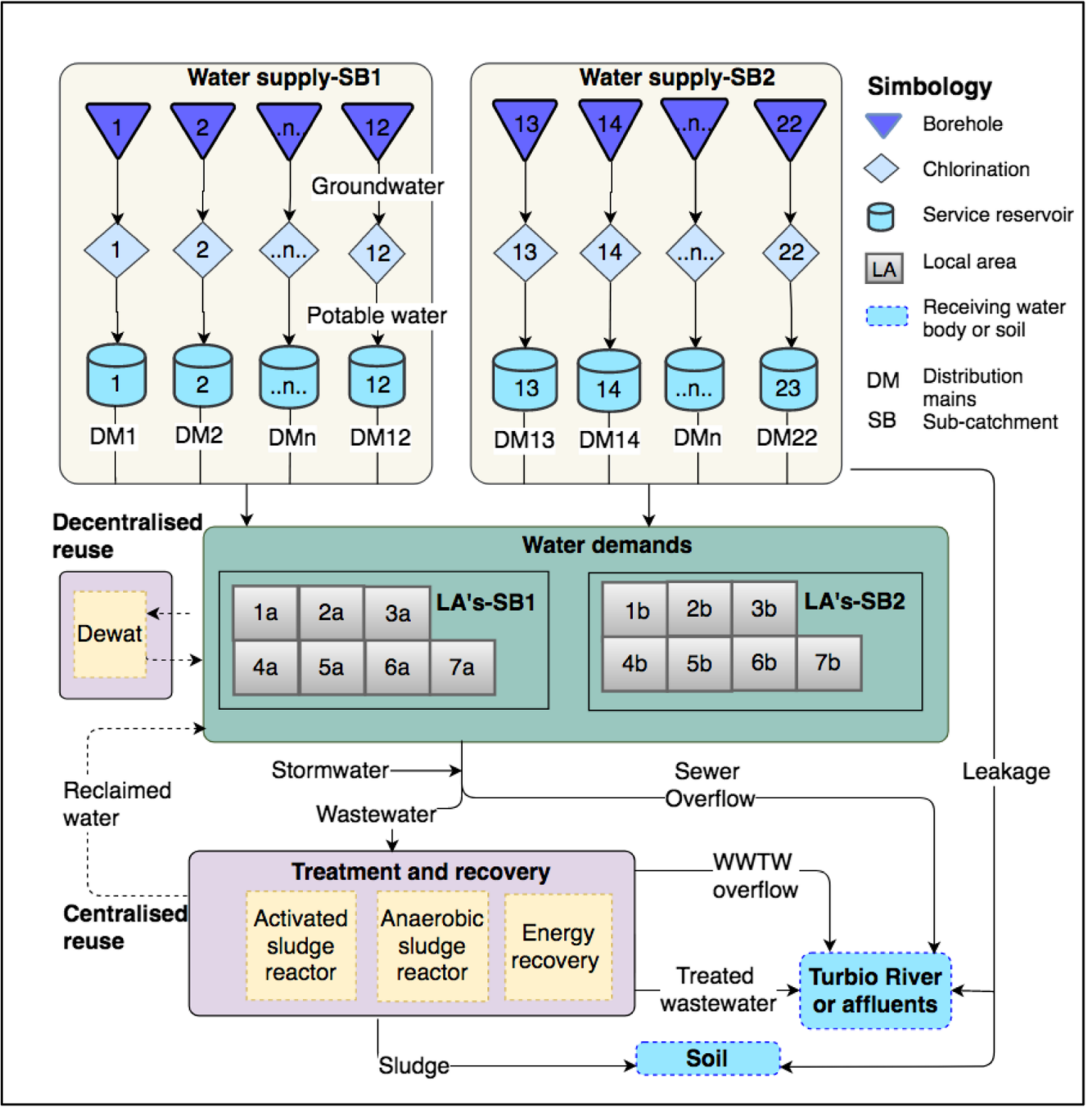
Schematic UWS layout in the Rincon cities, Mexico

The case study including both cities was considered to be the boundary of the urban area, i.e. the highest spatial level in WM2 (Landa-Cansigno et al. 2018). Each city represented one subcatchment (SB) and therefore the two subcatchments considered were SB1 (San Francisco del Rincon city or SFR) and SB2 (Purisima del Rincon city or PR). It was assumed that each subcatchment is made up of seven local areas with various sizes and specifications (Table 3). Each local area comprises a number of similar indoor areas (households), combined with industrial/commercial sectors and outdoor areas. Figure 3 presents the schematic layout of the UWS components in the case study.

**Table 3:** Specifications of the subcatchments (SBs) and local areas (LAs)

SB No.	No. of LAs	Area (ha)	Inhabitant	No. of households	Industrial demand (m^3^/day)	Irrigation demand (m^3^/day)
SB1 (SFR)	2	81	3458	781	120	15
	2	162	6917	1561	240	30
	2	323	13,835	3123	480	60
	1	485	20,750	4684	720	90
SB2 (PR)	2	62	2121	467	15	5
	2	123	4242	934	30	10
	2	246	8489	1870	60	20
	1	368	12,727	2803	90	30

The profile of various water demands detailed in Table 3 was calculated for each local area based on the annual water demand in Table 2 multiplied by the proportion of the area and inhabitants in the corresponding subcatchment. Irrigation demand (*WD*_i_) is calculated as

1 $$ W{D}_{\mathrm{i}}={A}_{\mathrm{i}}\times {I}_{\mathrm{f}}\times a\times 0.001\ \left(\raisebox{1ex}{${\mathrm{m}}^3$}\!\left/ \!\raisebox{-1ex}{$\mathrm{L}$}\right.\right) $$

where *A*_i_ is the area of irrigation (m^2^), *I*_f_ is the water per square metre in the area (5 L), and *a* is a correction factor assuming irrigation is undertaken once every 3 days (0.5). There is no available analysis for allocation of water demand to household appliance and fittings in the case study. Hence, it is assumed that the domestic water demand profile for all local areas includes 32% for toilet flushing, 22% for shower, 15% for washing machine, 15% for kitchens, 9% for hand basin, and 6% for irrigation including garden watering as recommended by Parker and Wilby (2013). No dishwasher is considered for indoor water demand in the UWS.

The calibration process involved a comparison between monthly observed and simulated water demand in 2015–2016. The first-year data was setup for calibration and the subsequent year for validation. The monthly water demand profiles per local area were adjusted during the calibration process, for example, assuming that there is no irrigation demand during July–September during the rainy season. Stormwater/wastewater subsystems were calibrated by adjusting the storage capacity, sewer network, perviousness and imperviousness, and rainfall-runoff coefficients in local areas. The calibration performance was evaluated by a number of statistical parameters, i.e. RSR, NSE, Pbias, and correlation coefficient that were compared against recommended values reported by Moriasi et al. (2007).

In addition to the data presented in Table 2, the main input data of the UWS are briefly described here and presented in Table 4. The daily and monthly water withdrawals were acquired for two continuous years of 2015 and 2016 for each city using primary data from local water utilities (SAPAP 2017; SAPAF 2017). Data on wastewater inflows and outflows were obtained for the period between 2015 and 2016 on a monthly scale (SITRATA 2017). Population data were obtained from the period 1990–2010 in various censuses from the National Statistical Institute of Mexico (INEGI). Population growth was estimated to be 1–3% using an arithmetic model (not shown here). Temperature and precipitation were obtained for the period between 1962 and 2011 from the ‘Guanajal’ station (CLICOM 2016). Vapour pressure and relative humidity from ‘Guanajuato Observatory station’ provided by the National Meteorological Service in Mexico (SMN 2017). The permeable area (sum of the green spaces and parks) data were obtained through the land use map database of the area (INEGI 2016). It was assumed that the runoff coefficient was 0.4 and infiltration coefficient was 0.5 for an urban area. Characterisation factors of GHG emissions and eutrophication for the production of chemicals used in the water/wastewater treatment were obtained from the CML World 2001 method and embodied energy values from cumulative energy demand method from the Ecoinvent3 database (Wernet et al. 2016). Fugitive emissions were obtained from the literature (see Table 4). It was considered that grid electricity in Mexico emits 0.458 kg CO_2_/kWh (SEMARNAT 2016). The study excludes the flow of materials used for the operational and maintenance phase of water distribution and sewer networks due to the lack of data in the case study.

**Table 4:** Main input data and key assumptions of the case study in WM2 model

Category	Data and source
Climatic data	Daily recorded data of rainfall, maximum, average, and minimum temperature from 1962–2011. Station Guanajal (− 101.8 W, 21.0 N; 1767 masl) (CLICOM 2016). Monthly average vapour pressure and relative humidity. Station Guanajuato Observatory (SMN 2017).
Demographic data	Total population per city XI Census 1990, XII Census 2000, XIII Census 2010; Population counts 1995, and 2005 from the National Institute of Statistics and Geography (INEGI 1990, 1995, 2000, 2005 and 2010).
Potable water sub-systems	Daily water extraction, energy consumption, and chemical use (2015–2016) in San Francisco and Purisima, Mexico.
Wastewater inflow and effluent	Daily flows in 2015–2016 (SITRATA 2017).
Sludge	Monthly flow production, biogas production, and disposal rate 2014–2016 (SITRATA 2017).
Chemical inputs in UWS	Chlorine gas (Cl_2_) produced through mercury cell process 0.0007 kg/m^3^_potable water_
	Sodium hypochlorite 15% solution (NaOCl), 0.0008 kg/m^3^_wastewater_ Iron chloride 40% in solution (FeCl_3_), 0.0054 kg/m^3^_wastewater_
GHG Emissions	Grid electricity in Mexico 0.458 kg CO_2_/kg (SEMARNAT 2016) Cl_2_ 1.28 kg CO_2_/kg (Wernet et al. 2016) NaOCl 0.96 kg CO_2_/kg (Wernet et al. 2016) FeCl_3_ 1.02 kg CO_2_/kg (Wernet et al. 2016).
Eutrophication	Electricity 4.6 × 10^5^ kgPO_4_eq/kWh (Wernet et al. 2016) Cl_2_ 0.002 kg PO_4_eq/kg (Wernet et al. 2016) NaOCl 0.0019 kg PO_4_eq/kg (Wernet et al. 2016) FeCl_3_ 0.0026 kg PO_4_eq/kg (Wernet et al. 2016).
Embodied energy	Cl_2_ 5.19 kWh/kg (Wernet et al. 2016) NaOCl 3.67 kWh/kg (Wernet et al. 2016) FeCl_3_ 3.92 kWh/kg (Wernet et al. 2016).
Fugitive emissions from sludge fertiliser	0.0143 kgCH_4_/dkg_sludge_ (Liu et al. 2013) 0.2 KgNH_3_-N/KgN_sludge_ (Foley et al. 2010) 0.00085 Kg N_2_O/dkg_sludge_ (Liu et al. 2013).

### Water reuse strategies

Nine hypothetical water reuse strategies from centralised and decentralised configurations (greywater and wastewater reuse) were considered for analysis and comparison. Three adoption or uptake proportions (20%, 50%, and 100%) were selected in consultation with key experts in the case study to evaluate potentials of using water reuse strategies with a wide range of uptake. From these nine strategies were defined and were composed of three centralised strategies (C20, C50, C100) that reuse treated wastewater of the WWTW, six decentralised strategies including three greywater ones (DG20, DG50, DG100) that reuse hand basin, washing machine, and shower effluents, and three wastewater ones (DW20, DW50, DW100) that reuse all domestic effluent. It was also assumed that the strategies will be implemented gradually in two time steps, at 10 and 20 years over a 30-year planning horizon. All strategies assumed that water reuse will be for toilet flushing, public irrigation, and industry. DG and DW strategies were assumed to use a membrane biological reactor (MBR) for greywater and wastewater treatment. Each household has a recycling tank with a capacity of 0.5 m^3^. The stormwater and domestic sewage quality were assumed to be according to a range of concentrations reported in the literature for five pollutants (BOD, COD, TSS, TN, and TP) given in Table 5.

**Table 5:** Pollutants concentration in mg/l selected for this study

Effluent	BOD	COD	TSS	TN	TP	Reference
Hand basin	101	208	20	5.1	3.3	Cardoso and Antunes (2017)
Kitchen sink	700	900	450	60	15	Antonopoulou et al. (2013); Li et al. (2009)
Washing machine	200	400	200	15	31	Vakil et al. (2014); Antonopoulou et al. (2013); Li et al. (2009)
Shower	200	300	200	15	5	Cardoso and Antunes (2017); Li et al. (2009)
Toilet	770	2000	1000	170	20	Molla (2013)
Runoff	10	70	60	1	1	Metcalf and Eddy (2003)
Industrial	1200	1500	1000	100	60	

The energy required for the transportation of treated wastewater in water reuse strategies was estimated based on the physical level difference and pipeline head losses between the WWTW, or DEWATS and six local area tanks (three for each subcatchment) where water reuse is transported and used. The transportation distances and level differences were estimated based on the digital elevation model and the land use maps in the case study (INEGI 2016). As such, distances were between 2 and 5 km and the level differences were between 20 and 50 m. Note that the energy required and the pipeline head loss based on the Hazen-Williams equation assume that recycled water has a continuous flow with velocity of 1 m/s and pump efficiency of 0.80.

The energy required for decentralised treatment was considered to be 0.93 kWh/m^3^, using reference values for a local area treatment facility consisting of screening, sand filter, MBR, and chlorine disinfection. Such values were assumed for a treatment facility of 5000 m^3^/day capacity according to Longo et al. (2016). Energy inputs per cubic metre of water reused are shown in Table 6.

**Table 6:** Energy demands in water reuse facilities in kWh per cubic metre of water reused

Type	Strategies	Treatment*	Transportation	Total
Centralised	C20	0.380	0.145	0.525
	C50	0.380	0.204	0.584
	C100	0.380	0.223	0.603
Decentralised domestic wastewater	DW20	0.930	0.123	1.050
	DW50	0.930	0.162	1.089
	DW100	0.930	0.221	1.148
Decentralised greywater	DG20	0.930	0.136	1.063
	DG50	0.930	0.181	1.108
	DG100	0.930	0.214	1.141

*Energy demands in centralised strategies corresponds to that in the WWTW

## Results and discussion

### Water-energy-pollution nexus

**Fig. 4:**
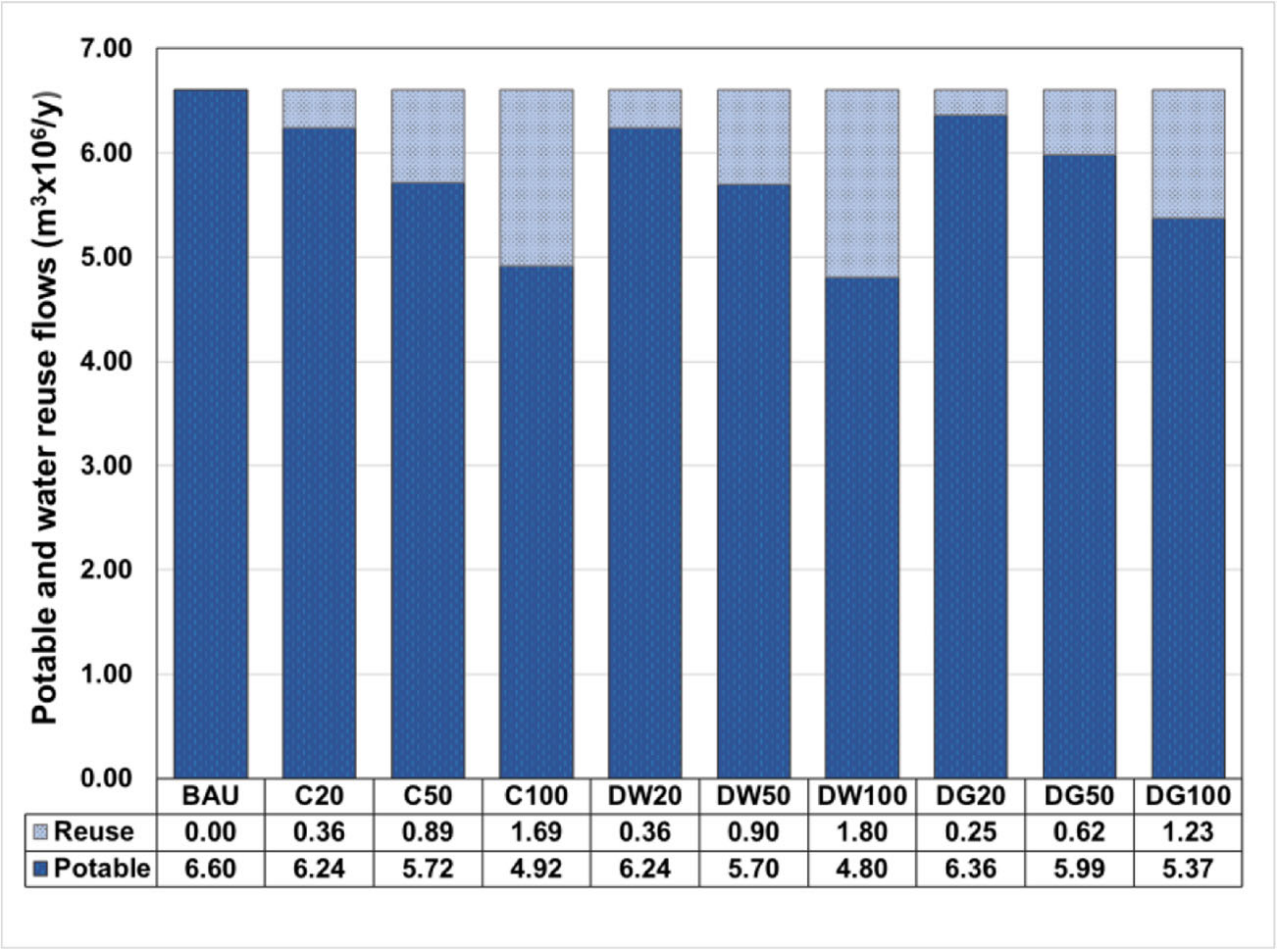
Annual average of potable water supply and water reused flows over the planning horizon in the nine strategies

The metabolism-based performance of the nine strategies (Table 4) was simulated for the case study by using WaterMet^2^ and was compared to the BAU with respect to the six assessment criteria (Table 1). The reliability of water supply over the planning horizon is almost 100% in the BAU (i.e. 99%). For water reuse strategies with adoption proportions equal to or above 50%, the total water demand is fully supplied (100%). When analysing an annual average of potable water supply in Fig. 4, strategies with higher adoption proportions can replace a larger proportion of potable water supply with water reuse. Both centralised and decentralised wastewater reuse (C and DW) seem to have relatively similar proportions of potential water reuse, which is higher than those in decentralised greywater reuse strategies (DG) in all uptake proportions.

**Fig. 5:**
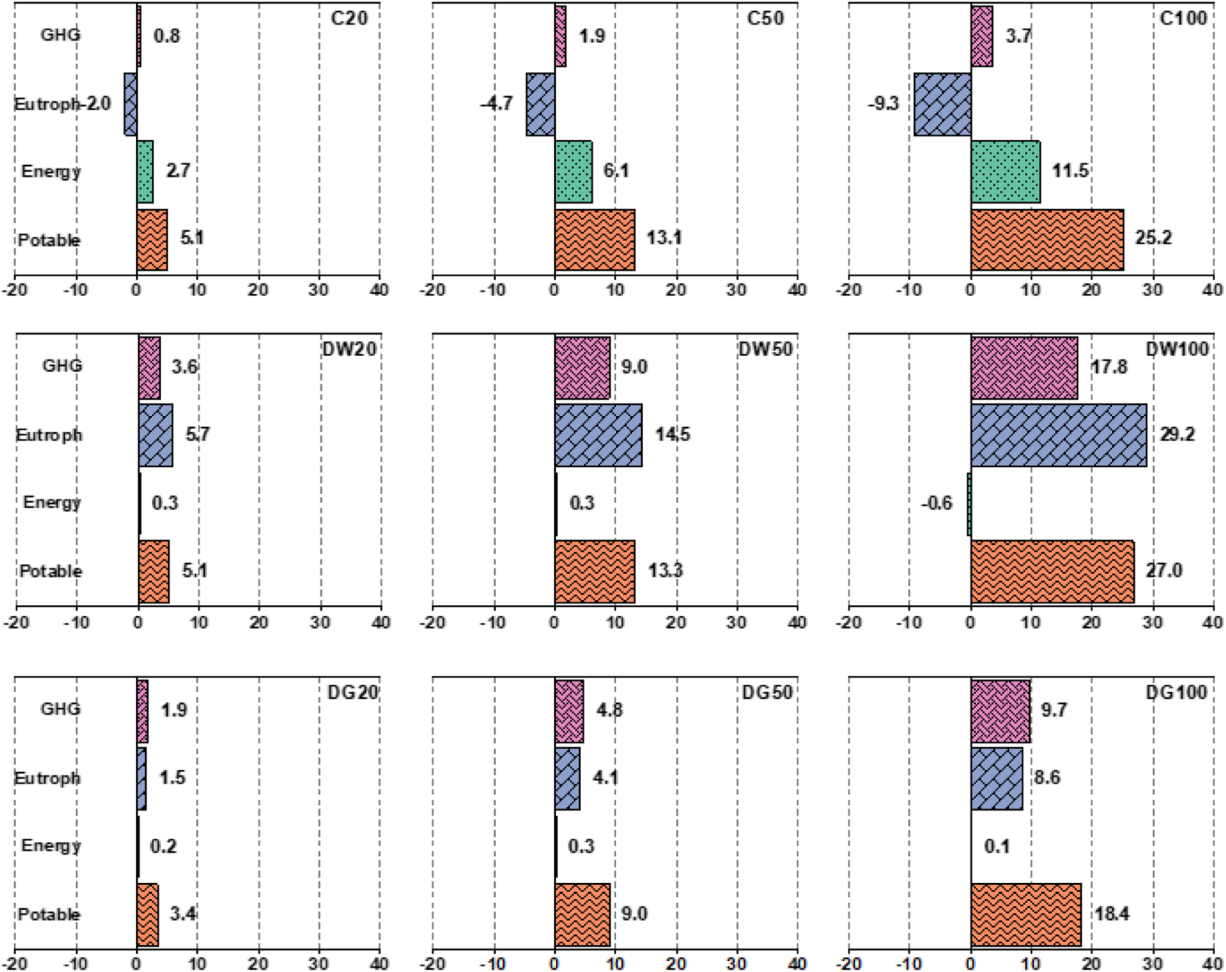
Percentages of changes of four KPIs in the nine strategies with respect to the BAU over the planning horizon, representing positive for reduction rates and negative for increase rates

Figure 5 shows the percentage of changes of four KPIs relative to the BAU for the nine strategies over the planning horizon. It is arguable that the most influential water reuse strategy is decentralised using domestic wastewater with 100% adoption proportion (DW100) which provides the greatest reductions for the potable water supply (27%), the EP (29.2%), and GHG emissions (17.8%), although the largest reduction in energy use (11.5%) is obtained through centralised water reuse using urban wastewater with 100% adoption proportion (C100). The application of various water reuse strategies is observed to have both positive and negative effects between the three WEP nexus elements. More specifically, all water reuse strategies would lead to saving potable water although it has relatively similar proportions in centralised and decentralised wastewater reuse strategies that are larger than those in decentralised greywater reuse. Reduction of GHG emissions also occurs for all water reuse strategies although their amounts for decentralised water reuse using domestic wastewater are much higher than other water reuse strategies (i.e., the reduction is almost two times greater in decentralised and five times greater in centralised greywater reuse strategies). This can be due to mainly decreasing the unused biogas in the UWS as a result of less domestic sewage being discharged into sewers/WWTWs and, also, less electricity being required for water withdrawals, treatment, and transportation within the water supply infrastructure.

Although all water reuse strategies would have positive impacts upon almost all KPIs in UWS, centralised strategies result in both positive and negative impacts simultaneously on KPIs (i.e. positive for potable water supply, GHG emissions and energy use and negative for EP). More specifically, the EP is significantly reduced in decentralised wastewater reuse compared to decentralised greywater reuse (for example, up to 29.2% in DW100 relative to 8.6% in DG100), whilst centralised wastewater reuse would increase (i.e. deteriorate) EP. This can be due to the fact that pollutants (mainly phosphorus) in centralised water reuse is recycling in the UWS and hence increases the load of contaminants into receiving water through the overflow of untreated effluent during heavy rainfall or the discharge of treated effluent. Such an effect does not exist in decentralised water reuse as domestic effluent into sewerage networks are reduced due to the diversion to DEWATS. This will be discussed in further detail later on in this section.

It is generally expected that greater changes of KPIs for each water reuse strategy take place in strategies with higher proportions of adoption. However, this is specifically inconsistent for energy use for all decentralised water reuse strategies in Fig. 5. More specifically, when comparing the total energy use of the water reuse strategies, it is evident that the highest energy savings are found to be related to centralised strategies (i.e. from 2.7% in C20 to 11.5% in C100), whilst decentralised strategies have almost negligible energy savings (0.1–0.3%) or even more energy use (i.e. − 0.6%. in DW100). The reason for these differences should be explored in the interactions between the caused and avoided energy in UWS components. The caused energy in UWS is that used in the potable water supply (i.e. for abstraction and treatment of raw water resources and distribution of potable water), centralised/decentralised water reuse facilities (i.e. for treatment and transportation), and avoided energy is the renewable energy generated in the WWTW. Table 7 shows a summary of annual energy for these four components in the UWS. As potable water is reduced in all water reuse strategies, it is expected that there will be less energy use for the water supply than that in the BAU, which is 0.753 kWh/m^3^. Caused energy for wastewater treatment and avoided energy for the generation of renewable electricity generation in the WWTW are relatively similar between the BAU and centralised strategies, although they are a little larger in centralised strategies due to 100% reliability levels. However, both caused and avoided energies are reduced in all decentralised strategies due to the reduction of domestic sewage discharging into wastewater systems. This reduction is less for decentralised greywater reuse as toilet water flushing is still discharged into wastewater systems. Under the assumptions made for energy demand in water reuse facilities in this case study, the centralised facilities are more energy efficient than the decentralised ones (Table 6). As a result, although all water reuse strategies would lead to reduced energy use in the water supply, the decentralised strategies show almost no change in total net energy use (Table 7 and Fig. 5). Having said this, it should be noted that decentralised facilities are not necessarily more energy intensive. Hence, more energy efficient decentralised water reuse technologies (unlike energy intensive ones such as MBR) should be analysed to achieve an improved energy performance in the UWS. In addition, decentralised facilities reduce renewable energy generation in WWTW and this can have a negative impact on the energy performance of decentralised strategies. In this case study, however, the contribution of renewable energy in total energy use is almost negligible (< 1%) and hence no sensible change in the net energy can be envisaged in different strategies.

**Table 7:** Annual energy use/generation in the main UWS components; note that all except renewable electricity are expressed in kWh per cubic metre of water demand

Strategy	Total net energy (kWh/m^3^)	Energy use			Energy generation
		Water supply (kWh/m^3^)	Wastewater treatment (kWh/m^3^)	Reuse facilities* (kWh/m^3^)	Total renewable electricity (kWh)
BAU	1.152	0.753	0.399	0.000	190.74
C20	1.121	0.713	0.400	0.008	191.23
C50	1.082	0.654	0.400	0.027	191.31
C100	1.020	0.563	0.400	0.057	191.31
DW20	1.149	0.713	0.379	0.057	181.22
DW50	1.148	0.652	0.347	0.148	166.22
DW100	1.159	0.551	0.295	0.313	141.07
DG20	1.150	0.724	0.385	0.040	184.36
DG50	1.149	0.681	0.364	0.103	174.17
DG100	1.151	0.609	0.328	0.213	156.99

*For centralised strategies, energy of reused facilities contains only transportation energy as the treatment energy in centralised facilities is included in wastewater treatment

**Fig. 6:**
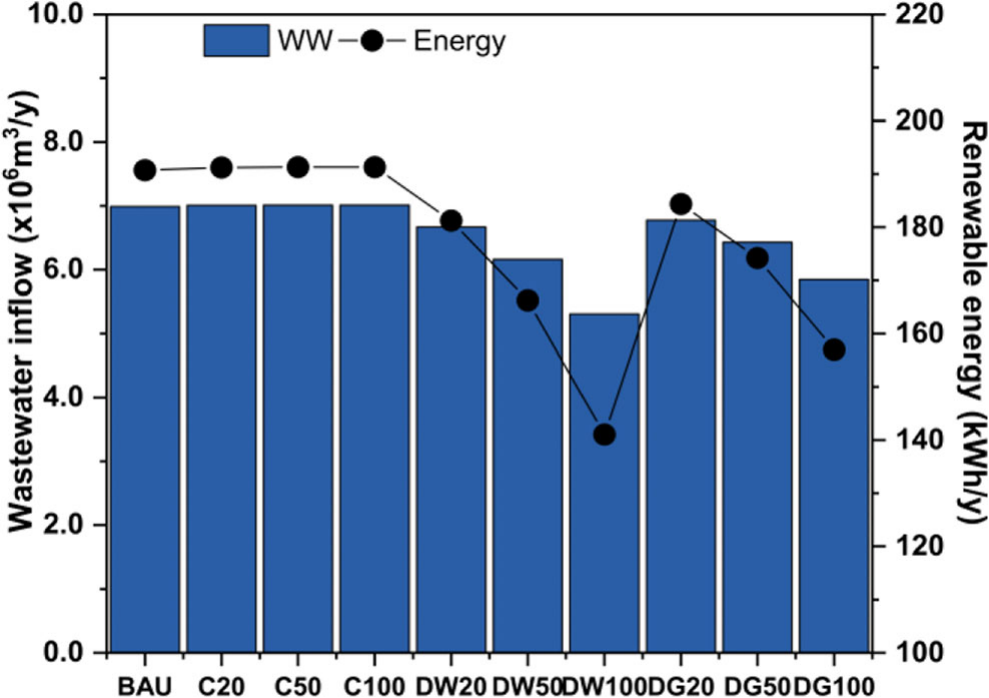
Wastewater inflow vs renewable energy of the WWTW in the BAU and nine strategies

The electricity required for the UWS operation in the case study is supplied from the grid and less than 1% is generated onsite by the biogas produced in the WWTW. Grid electricity in Mexico is mainly sourced by fossil fuels, 87% gas and coal and 13% other sources (Santoyo-Castelazo et al. 2014). As the wastewater inflow to the WWTW is reduced due to the implementation of decentralised strategies, the proportion of renewable energy produced is reduced in these strategies, as shown in Fig. 6. In particular, DW100 would experience the highest reduction of renewable energy generation (i.e. 26%) as the wastewater inflow to the WWTW decreases by 25% relative to the BAU. On the other hand, this proportion is not affected in centralised strategies as wastewater inflows remain equal compared with the BAU. Although the total amount of renewable energy generation in all strategies is minor compared with considerable fossil-based electricity from the grid, they are important since producing more clean energy is in agreement with international commitments for climate change mitigation and adaptation.

**Fig. 7:**
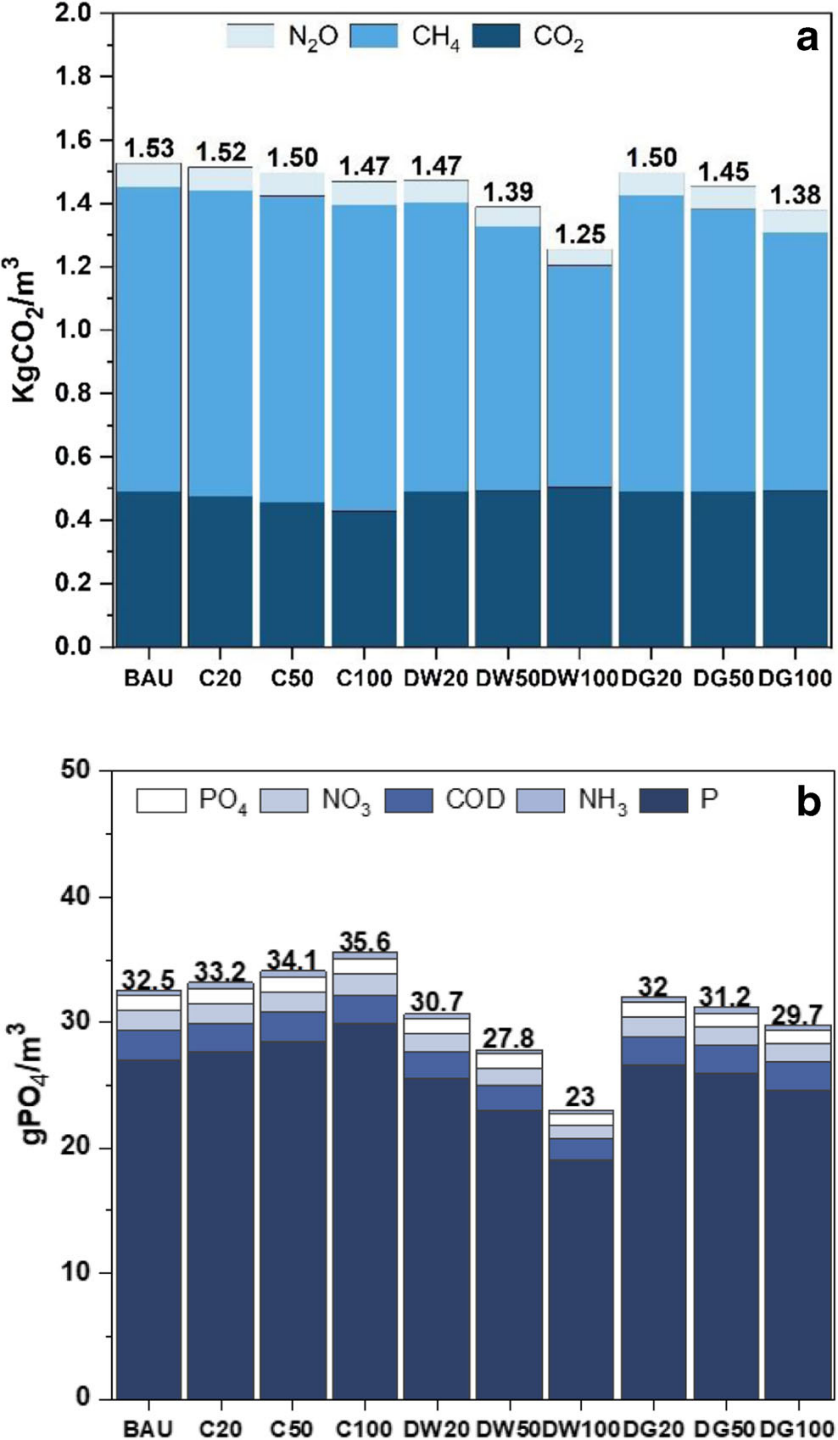
Constituents of (**a**) GHG emissions and (**b**) eutrophication potential (EP) in the BAU and nine strategies; both are expressed as annual average of CO_2_/PO_4_ per cubic metre of water demand

Figure 7 a shows the contribution of elements constituting GHG emissions and the EP in the UWS for the BAU and nine strategies. These elements are three gases (CO_2_, CH_4_, and N_2_O) for GHG emissions and P, NH_3_, COD, NO_3_, and PO_4_ emissions to water for eutrophication. As for GHG emissions, CO_2_ emissions are the result of direct (i.e. fossil fuel and electricity) and indirect (i.e. chemicals) emissions in all UWS components, while CH_4_ and N_2_O are emitted from the WWTW. As can be seen, CH4 is the major component (60%), contributing to GHG emissions, compared to CO_2_ (33%) in the BAU. CH_4_ in the WWTW is resulted from the unused biogas that is burned and released into the atmosphere. Consequently, those strategies that result in reducing wastewater inflow to the WWTW (i.e. decentralised strategies as described earlier) would lead to greater reduction in GHG emissions. As a result, the highest reduction can be obtained from decentralised water reuse using wastewater with a 100% adoption proportion (DW100) which reduces GHG emissions by 17.8%. Therefore, special attention should be paid to increasing the potential biogas utilisation to reduce GHG emissions in the UWS.

A relatively similar trend exists for the impact of the water reuse strategies on the EP in Fig. 7b. In particular, the main component of the EP is made of P which is the result of effluents discharged into receiving water from either treated effluent of the WWTW, untreated effluents of sewer networks, or the WWTW. Similarly, decentralised strategies would reduce the wastewater inflows in both sewer networks and WWTW and hence the EP would reduce significantly. On the other hand, centralised strategies return the treated effluent of the WWTW to the UWS to be used for non-potable water demands (i.e. toilet flushing and irrigation). Although the pollutants in this treated effluent that is replaced with potable water are within acceptable limits for non-potable uses, the recycling effluent clearly adds more pollutant to the UWS than the BAU. Once this additional load of pollutants is converted into wastewater, specifically in toilet flushing, the overall load of pollutants can increase in sewer networks and the WWTW. Consequently, the EP can increase slightly as seen in Fig. 7b for all centralised strategies.

## Conclusions

The long-term performance assessment of a number of centralised and decentralised water reuse strategies in the integrated UWS was conducted in this paper by using an integrated framework of the WEP nexus and urban water metabolism. The water reuse strategies considered using both greywater at local scale and domestic/urban wastewater at local/urban scale. The modelling approach of urban water metabolism was considered to first integrate all main UWS components of the water supply, stormwater, and wastewater subsystems before incorporating the influence of the intervention strategies on other components of the urban water cycle.

From the WEP nexus perspective, decentralised water reuse strategies using domestic wastewater were found in this study to perform the best with respect to potable water saving, reductions of eutrophication and GHG emissions, while centralised strategies can provide the largest savings of energy use in the UWS. Having said this, centralised strategies can deteriorate eutrophication potential due to the discharge of more pollutants into the urban water cycle. The results show that the interaction between the WEP nexus elements can be quantified as a result of the metabolic performance simulation of integrated UWS. Consequently, the assessment of water reuse strategies with respect to WEP nexus criteria can unveil the direct and indirect influences between the nexus elements (i.e. water, energy, and pollution) that are either difficult to recognise or unexpected due to the complexity of the integrated UWS. The opposite influences can occur due to the complex and indirect interaction that might exist between the UWS components and overall system.

Although the current methodology used a typical real-world case study to explore the capabilities of the suggested framework, the findings obtained in this paper cannot be generalised for other UWS. For example, the adoption/uptake percentages of water reuse options must be tailored based on the socio-economic factors in the UWS. Hence, more test cases should be conducted on other real-world case studies in order to extract some general outcome with respect to the water-energy-pollution nexus for water reuse strategies.
